# HTRA1 interacts with SLC7A11 to modulate colorectal cancer chemosensitivity by inhibiting ferroptosis

**DOI:** 10.1038/s41420-024-01993-6

**Published:** 2024-05-13

**Authors:** Weiwei Liu, Chaoqun Liu, Jun Xiao, Cheng Qian, Zhilin Chen, Wandie Lin, Yujie Zhang, Jianghua Wu, Rui Zhou, Liang Zhao

**Affiliations:** 1 Department of Pathology, Shunde Hospital, Southern Medical University, Foshan, China; 2 Department of Pathology & Guangdong Province Key Laboratory of Molecular Tumor Pathology, School of Basic Medical Sciences, Southern Medical University, Guangzhou, China; 3 Department of Pathology, Nanfang Hospital, Southern Medical University, Guangzhou, China

**Keywords:** Colorectal cancer, Cancer therapeutic resistance

## Abstract

Chemotherapy is an important therapuetic strategy for colorectal cancer (CRC), but chemoresistance severely affects its efficacy, and the underlying mechanism has not been fully elucidated. Increasing evidence suggests that lipid peroxidation imbalance-mediated ferroptosis is closely associated with chemoresistance. Hence, targeting ferroptosis pathways or modulating the tolerance to oxidative stress might be an effective strategy to reverse tumor chemoresistance. HtrA serine protease 1 (HTRA1) was screened out as a CRC progression- and chemoresistance-related gene. It is highly expressed in CRC cells and negatively correlated with the prognosis of CRC patients. Gain- and loss-of-function analyses demonstrated a stimulatory role of HTRA1 on the proliferation of CRC cells. The enrichment analysis of HTRA1-interacting proteins indicated the involvement of ferroptosis in the HTRA1-mediated chemoresistance. Moreover, electron microscope analysis, as well as the ROS and MDA levels in CRC cells also confirmed the effect of HTRA1 on ferroptosis. We also verified that HTRA1 could interact with SLC7A11 through its Kazal structural domain and up-regulate the expression of SLC7A11, which in turn inhibited the ferroptosis and leaded to the chemoresistance of CRC cells to 5-FU/L-OHP. Hence, we propose that HTRA1 may be a potential therapeutic target and a prognostic indicator in CRC.

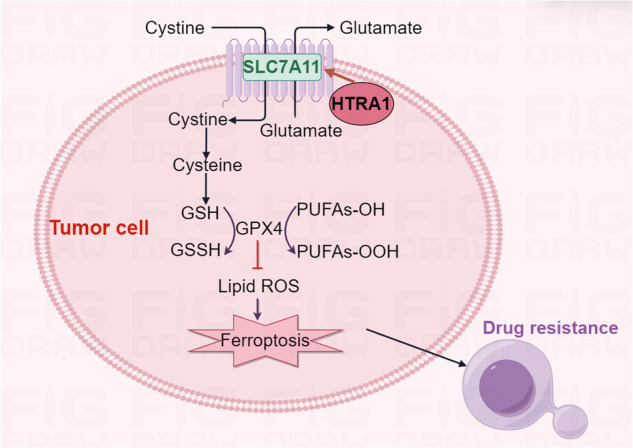

## Introduction

Colorectal cancer (CRC) is one of the most common malignant tumors in the digestive system, which seriously affects human health. According to the global cancer statistics released by the International Agency for Research on Cancer (IARC) in 2020, the morbidity and mortality of CRC ranked the third and the second highest among malignant tumors, respectively [1]. Chemotherapy is not only used as an adjuvant postoperative treatment for early-stage CRC patients, but also considered as the mainstay of comprehensive treatment for intermediate and advanced CRC [2]. However, chemoresistance of CRC cells seriously affects the therapeutic efficacy and associates with a poor prognosis of CRC patients [3]. Increasing evidence suggests that oxidative stress defense mediated by the imbalance of intracellular reactive oxygen species (ROS) in tumor cells is closely associated with tumor chemoresistance [4]. Ferroptosis, discovered and characterized by Dixon et al. in 2012, is a new form of cell death distinguished from apoptosis, necrosis and autophagy, which is essentially caused by iron-dependent lipid ROS accumulation [5]. Solute carrier family 7 member 11 (SLC7A11/xCT) is a key functional subunit of the Xc-system, and acts as a cystine/glutamate antitransporter protein that transports extracellular cystine into the cells. The promotion of cystine uptake leads to a rapid conversion of cystine to cysteine, which is a vital precursor for glutathione (GSH) synthesis [6]. Subsequently, glutathione peroxidase 4 (GPX4) utilizes GSH as a co-factor to reduce lipid hydroperoxides to lipohydrols, thereby protecting cells from lipid peroxidation-induced and iron-dependent cell death [7]. SLC7A11 is generally upregulated in tumor cells, particularly in those chemotherapy/radiotherapy-resistant tumor cells [6, 8]. Genetic albation of SLC7A11 or pharmacological inhibition of its activity can reduce the synthesis GSH, which leads to the production and accumulation of lipid peroxidation products, and ultimately induces ferroptosis in cancer cells [6, 9–12].

HtrA serine proteinase 1 (HTRA1), also known as DegP in bacteria, belongs to the HTRA family of serine proteases, which are serine proteases with heat shock protein properties. HTRA1 contains four functional domains, including PDZ domain, serine protease domain, insulin-1ike growth factor binding protein domain, and Kazal structural domain [13]. HTRA1 is aberrantly expressed in various tumors. A reduced expression of HTRA1 was detected in several malignant tumors including ovarian carcinoma, melanoma [14] and endometrial carcinoma [15], and it is considered as a tumor suppressor gene [16]. It was also identified as a oncogene in other cancers, such as gastric cancer, for its elevated expression [17]. Hence, HTRA1 obtains a bi-directional regulation potential in different tumor cells. However, recent studies mainly focus on the effects of HTRA1 on tumor progression, and its functional mechanism in CRC has not been clarified yet.

In this study, we identified the role of HTRA1 in CRC chemoresistance, and revealed its critical role in reducing the lipid peroxidation-induced ferroptosis. We found that HTRA1 could interact with SLC7A11 to promote cystine uptake, and thus reduce the accumulation of lipid peroxidation products. We also studied its effect on chemoresistance by targeting HTRA1 and ferroptosis. Our results will reveal the functional mechanism of HTRA1 in CRC chemoresistance and demonstrate the therapeutic potential of targeting HTRA1 in the clinical treatment of CRC.

## Results

### Overexpression of HTRA1 is associated with malignant progression of CRC

**Fig. 1:**
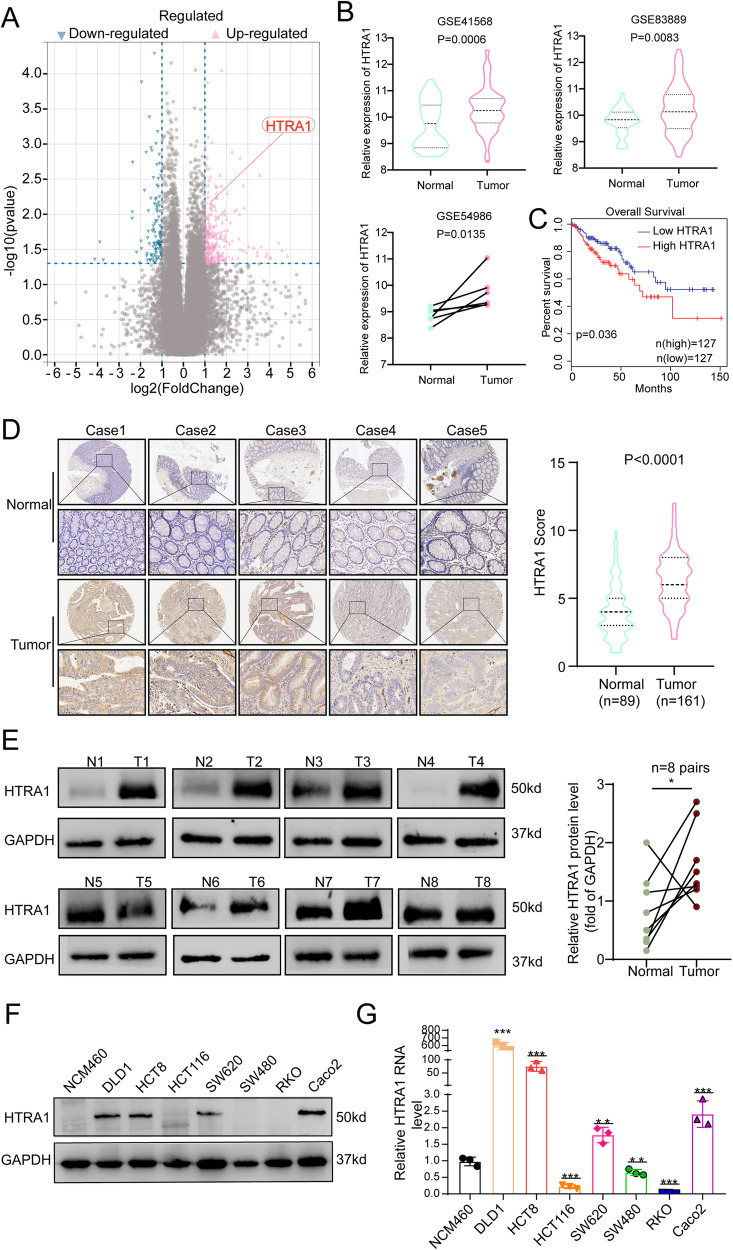
Overexpression of HTRA1 is associated with CRC malignant progression. **A** Volcanogram shows progression-related DEGs between normal colorectal mucosa and CRC tissues. **B** The expression of HTRA1 in normal and CRC tissues analyzed using the GEO database. **C** Kaplan–Meier survival curves show the relationship between HTRA1 expression and the overall survival of CRC patients. **D** IHC analyses show the expression of HTRA1 in CRC tissues (T) and normal mucosa (N) of five representative CRC tissue samples. **E** Western blot analyses show the expression of HTRA1 in fresh CRC tissues (T) and the adjacent non-tumor tissues (N), **F**, **G** Western blot and real-time q-PCR demonstrate the expression of HTRA1 in CRC cells. All the data represent the mean ± SD of at least three independent experiments. *P < 0.05, **P < 0.01, and ***P < 0.001. Differences were tested using a two-tailed, paired (**B**, **E**) and unpaired (**B**, **D**, **G**) Student’s *t* test.

HTRA1, also known as high temperature requirement factor 1, was successfully screened out as a CRC progression-related gene through analyzing our previously published high throughput mRNA microarray datasets, which contains 8 pairs of human CRC tissue samples at stage I-II and stage III-IV (Fig. 1A, GSE113296). Although HTRA1 ranked the 26th in the list of differential expression genes, but after excluding those without prognostic significance, and those well-studied in cancers, HTRA1 is the one that best meet our requirement. To investigate the role of HTRA1 in CRC progression, we analyzed the expression of HTRA1 in Gene Expression Omnibus (GEO) datasets, including GSE41568, GSE83889 and GSE54986, and the abnormally elevated expression of HTRA1 was detected in CRC tissues compared with the normal intestinal mucosa (Fig. 1B). The Cancer Genome Atlas (TCGA) database was used to study the correlation between HTRA1 expression and the prognosis of CRC patients (Fig. 1C and Supplementary Fig. S1). In addition, IHC staining of the clinical CRC samples from our affiliated hospitals also showed that HTRA1 was overexpressed in CRC tissues compared with paired intestinal mucosa (Fig. 1D). Consistently, western blot analyses showed that HTRA1 expression was higher in fresh CRC tissues than in matched non-tumor tissues (Fig. 1E). We also investigated the expression of HTRA1 in normal colon cells and seven different CRC cell lines, and found that HTRA1 was highly expressed in DLD1, HCT8, and SW620 cells compared with normal human colonic epithelial NCM460 cells at both transcriptional and translational levels (Fig. 1F, G).

### HTRA1 promotes the proliferation and chemoresistance of CRC cells

**Fig. 2:**
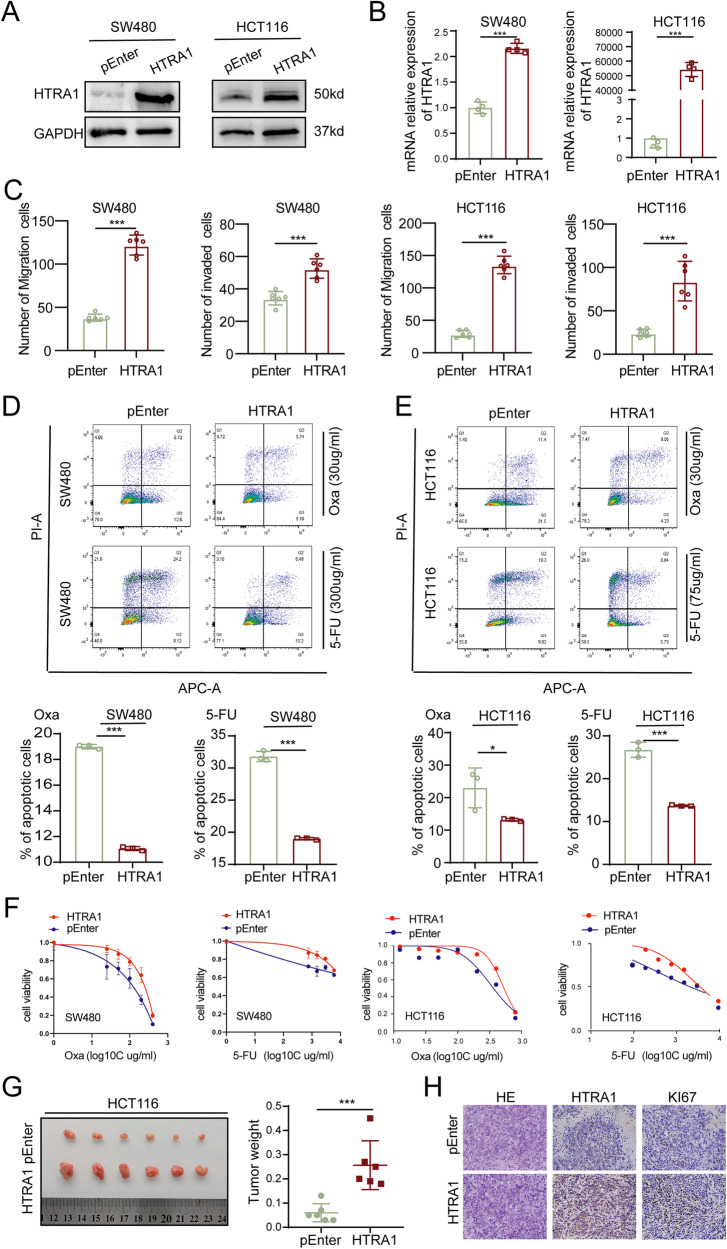
Overexpression of HTRA1 promotes the proliferation and chemoresistance of CRC cells. **A**, **B** Western blot and Real-time q-PCR assays demonstrate the successful construction of HTRA1 overexpression CRC cells. **C** Transwell assays show the effect of HTRA1 on the invasive and migratory ability of SW480 and HCT116 cells. **D**, **E** Flow cytometry analyses show the effect of HTRA1 on the apoptosis of CRC cells after 5-FU and Oxa treatment. **F** The effects of HTRA1 on the cell viability of CRC cells after 5-FU and Oxa treatment. **G** Subcutaneous tumors formed in nude mice by control and HTRA1 overexpression HCT116 cells. Right panel shows the weight of subcutaneous tumors. **H** IHC staining was performed to detect the expression of HTRA1 and Ki67 in the indicated subcutaneous tumors in nude mice. All the data represent the mean ± SD at least three independent experiments. **P < 0.01 and ***P < 0.001. Differences were tested using an unpaired two tailed Student’s *t* test (**B**–**E**, **G**).

To investigate the biological effects of HTRA1 on CRC cells, we transfected pEnter-HTRA1 vectors into SW480 and HCT116 cells, and successfully obtained HTRA1 overexpression CRC cells (Fig. 2A, B). Transwell assays demonstrated a stimulatory effect of HTRA1 on the invasion and migration of CRC cells (Fig. 2C and Supplementary Fig. S4A). Flow cytometry assays demonstrated a decreased apoptosis in HTRA1 overexpression SW480 and HCT116 cells compared with CRC cells transfected with the pEnter vector (Fig. 2D, E). Moreover, HTRA1 could increase the viability of 5-FU/L-OHP-treated SW480 and HCT116 cells (Fig. 2F). The subcutaneous tumors formed by HTRA1 overexpression cells were larger in size and heavier in weight than that formed by control cells (Fig. 2G). IHC staining for the Ki-67 index also confirmed that HTRA1 could enhance the growth of subcutaneous tumors formed by CRC cells (Fig. 2H).

**Fig. 3:**
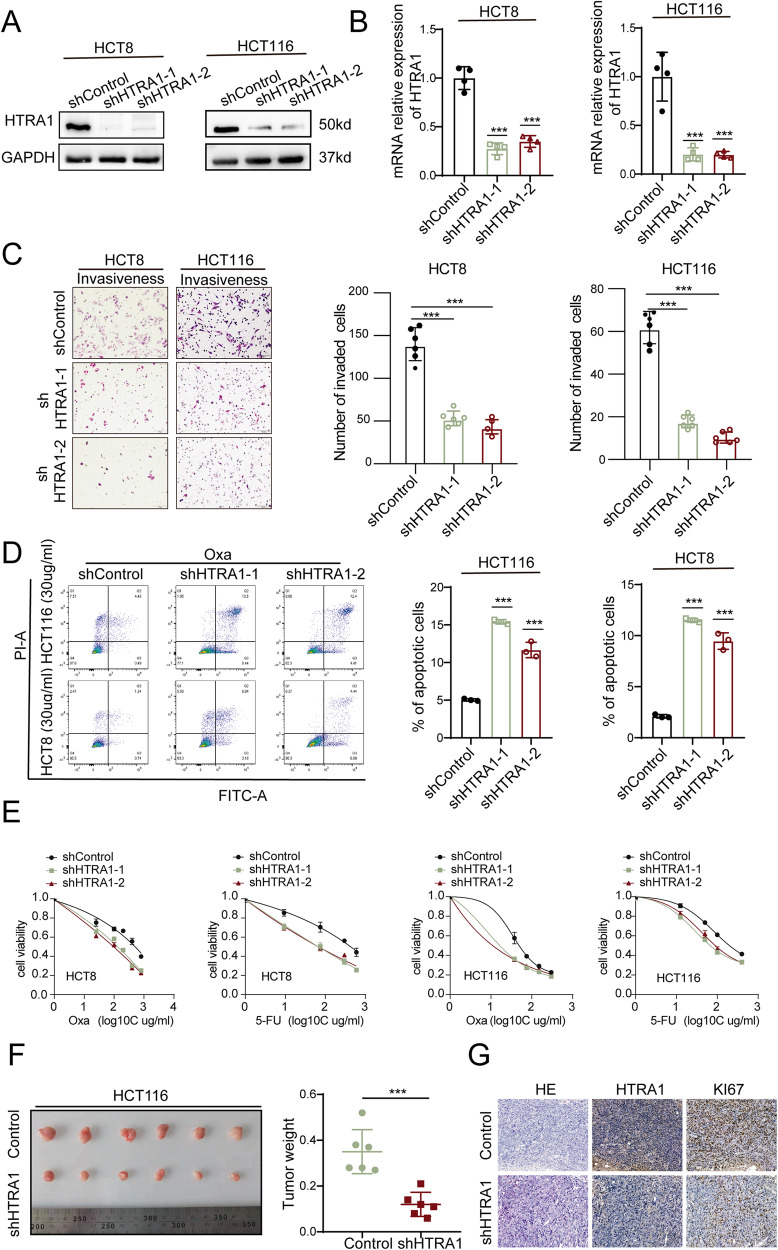
Knockdown of HTRA1 inhibits the proliferation and chemoresistance of CRC cells. **A**, **B** Western blot and Real-time q-PCR assays demonstrate the successful construction of HTRA1 knockdown CRC cells. **C** Transwell assays show that inhibiting the expression of HTRA1 suppresses the invasive ability of HCT8 and HCT116 cells. **D** Flow cytometry analyses show that inhibiting the expression of HTRA1 stimulates the apoptosis of CRC cells after 5-FU and oXA treatment. **E** Inhibiting the expression of HTRA1 suppresses the cell viability of CRC cells after 5 -FU and oXA treatment. **F** The subcutaneous tumors formed in nude mice by control and HTRA1 knockdown HCT116 cells. The right panel shows the weight of subcutaneous tumors. **G** IHC staining was performed to detect the expression of HTRA1 and Ki67 in the indicated subcutaneous tumors in nude mice. All the data represent the mean ± SD at least three independent experiments. **P < 0.01 and ***P < 0.001. Differences were tested using an unpaired two tailed Student’s *t* test (**B**–**D**, **F**).

Then, two shRNAs were used to inhibit the expression of HTRA1, and the intefering efficiency was confirmed by both western blot and real-time qPCR (Fig. 3A, B). Transwell assays demonstrated that knockdown of HTRA1 attenuated the invasive and migratory abilities of HCT8 and HCT116 CRC cells (Fig. 3C and Supplementary Fig. S4B). Flow cytometry assays showed that knockdown of HTRA1 increased the apoptosis of HCT8 and HCT116 CRC cells (Fig. 3D and Supplementary Fig. S4C). Drug sensitivity assays also confirmed that the interference of HTRA1 could increase the sensitivity of CRC cells to 5-FU/L-OHP (Fig. 3E). Moreover, the interference of HTRA1 suppressed the growth (Fig. 3F) and Ki-67 index of subcutaneous tumors formed by HCT116 cells (Fig. 3G). In conclusion, the above results suggest that HTRA1 can promote the proliferation and chemoresistance of CRC cells both in vitro and in vivo.

### HTRA1 suppresses the ferroptosis of CRC cells

**Fig. 4:**
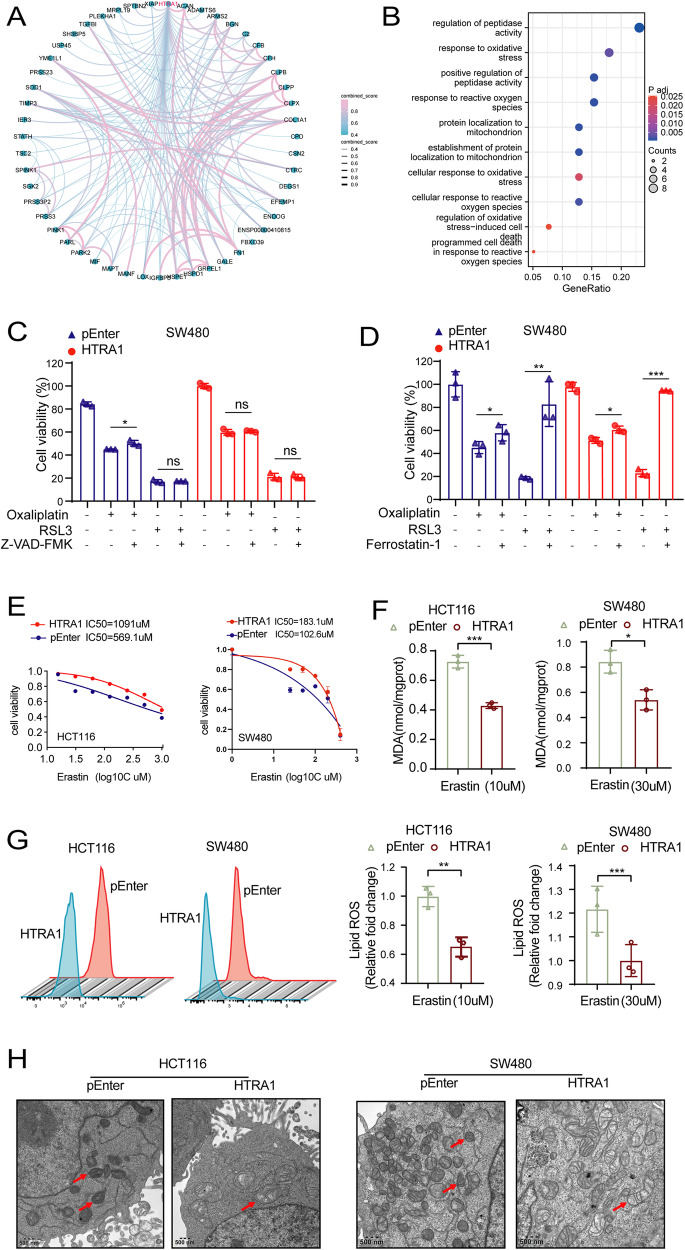
HTRA1 suppresses the ferroptosis in CRC cells. **A** The HTRA1-interacting proteins were screened out using STRING database. **B** GO enrichment analysis was performed based on the HTRA1-interacting genes. **C**, **D** The effects of apoptosis inhibitor and ferroptosis inhibitor on the growth of SW480 cells after oXA and RSL3 treatment. **E** The effect of Erastin on the cell viability of HTRA1 overexpression CRC cells. **F** The MDA level was detected in HTRA1 overexpression CRC cells after Erastin treatment for 24 h. **G** Flow cytometry analyses were used to evaluate the lipid ROS levels in HTRA1 overexpressing CRC cells. **H** Transmission electron microscopy was used to detect the effects of HTRA1 on the morphology related to ferroptosis in CRC cells. All the data represent represent the mean ± SD at least three independent experiments. **P < 0.01 and ***P < 0.001. Differences were tested using an unpaired two tailed Student’s *t* test (**C**, **D**, **F**, **G**).

To further explore the mechanism underlying HTRA1-mediated CRC progression, we predicted fifty HTRA1-interacting proteins from the PPI network based on the STRING database (Fig. 4A). The pathway enrichment analysis of HTRA1-interacting proteins revealed that redox-responsive pathways were significantly enriched (Fig. 4B). Therefore, we speculated whether HTRA1 could enhance CRC progression by affecting ferroptosis. To exclude the influence of HTRA1 on other types of cell death triggered by chemotherapy, the apoptosis inhibitor Z-VAD-FMK, and ferroptosis inhibitor Ferrostatin-1 were used to rescue cell death. As shown in Fig. 4C, Z-VAD-FMK could moderately inhibit the L-OPH-induced cell death, but not the RSL3-induced cell death of CRC cells. However, Ferrostatin-1 (Ferr-1) could inhibit both L-OPH and RSL3-induced cell death (Fig. 4D). To examine the role of HTRA1 on ferroptosis, CRC cells were pre-treated with RSL/Erastin for 24 h to raise the baseline ferroptosis level before cell viability and MDA level detection. It was shown that overexpression of HTRA1 could enhance the viability of Erastin/RSL3-treated HCT116 and SW480 cells (Fig. 4E and Supplementary Fig. S5A). To explore the effect of HTRA1 on ferroptosis, we measured the intracellular MDA levels, an oxidative stress marker, as well as the ROS levels in control and HTRA1 overexpressing HCT116 and SW480 cells. The results showed that MDA levels in HTRA1 overexpressing CRC cells was significantly reduced compared with that in control CRC cells (Fig. 4F and Supplementary Fig. S5B), and so was the the ROS levels (Fig. 4G and Supplementary Fig. S5C). Meanwhile, TEM assay showed that the mitochondrial cristae were reduced, the outer mitochondrial membranes were ruptured and crumpled, and the mitochondria were darkly stained in the control group, compared with that in the HTRA1 overexpression group (Fig. 4H).

**Fig. 5:**
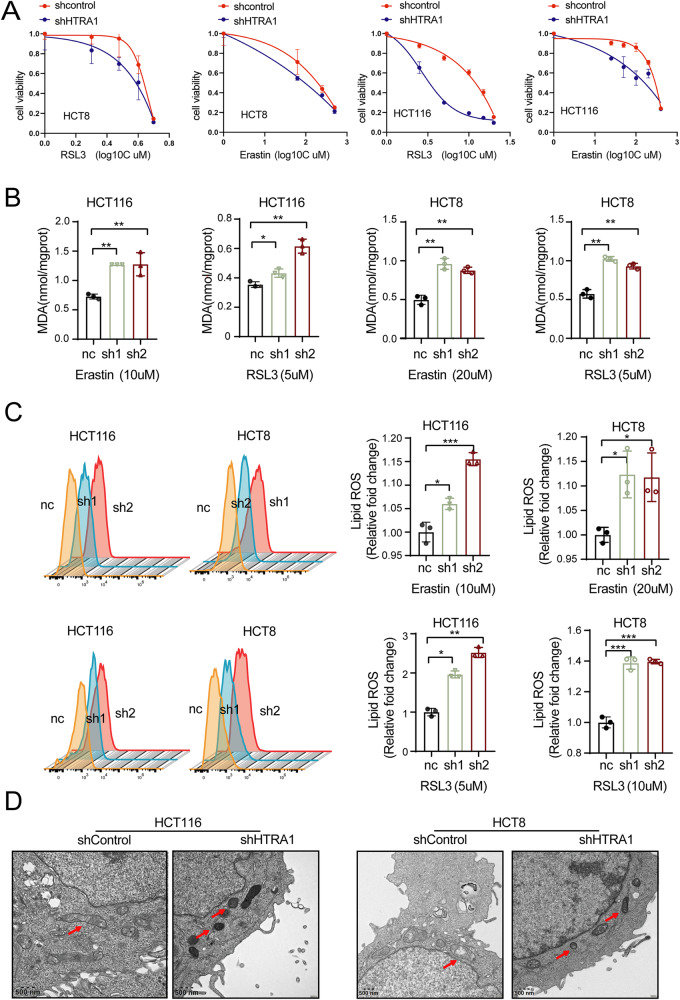
Knockdown of HTRA1 induces the ferroptosis in CRC cells. **A** Cell viability analyses show the effect of HTRA1 on the Erastin and RSL3 treated CRC cells by interfering HTRA1. **B** The MDA level was detected in control and HTRA1 knockdown CRC cells after Erastin treatment for 24 h. **C** Flow cytometry analyses were used to evaluate the lipid ROS levels in control and HTRA1 knockdown CRC cells. **D** Transmission electron microscopy was used to detect the effects of HTRA1 knockdown on the morphology related to ferroptosis in CRC cells by interfering HTRA1. All the data represent the mean ± SD at least three independent experiments. **P < 0.01 and ***P < 0.001. Differences were tested using an unpaired two tailed Student’s *t* test (**B**, **C**).

To further confirm the role of HTRA1 in ferroptosis, we knocked down HTRA1 to explore its effect on ferroptosis-related factors. Knockdown of HTRA1 reduced the viability of Erastin/RSL3-treated HCT116 and SW480 cells (Fig. 5A), but increased the intracellular MDA and ROS levels in HCT8 and HCT116 cells (Fig. 5B, C). In addition, TEM assay showed the reduced mitochondrial cristae, ruptured and wrinkled outer mitochondrial membranes, as well as dark stained mitochondria in HTRA1 knockdown HCT8 and HCT116 cells, compared with the control group (Fig. 5D). Taken together, our data suggest that HTRA1 plays an important role in regulating ferroptosis in CRC cells.

### HTRA1 interacts with SLC7A11 and promotes its expression

**Fig. 6:**
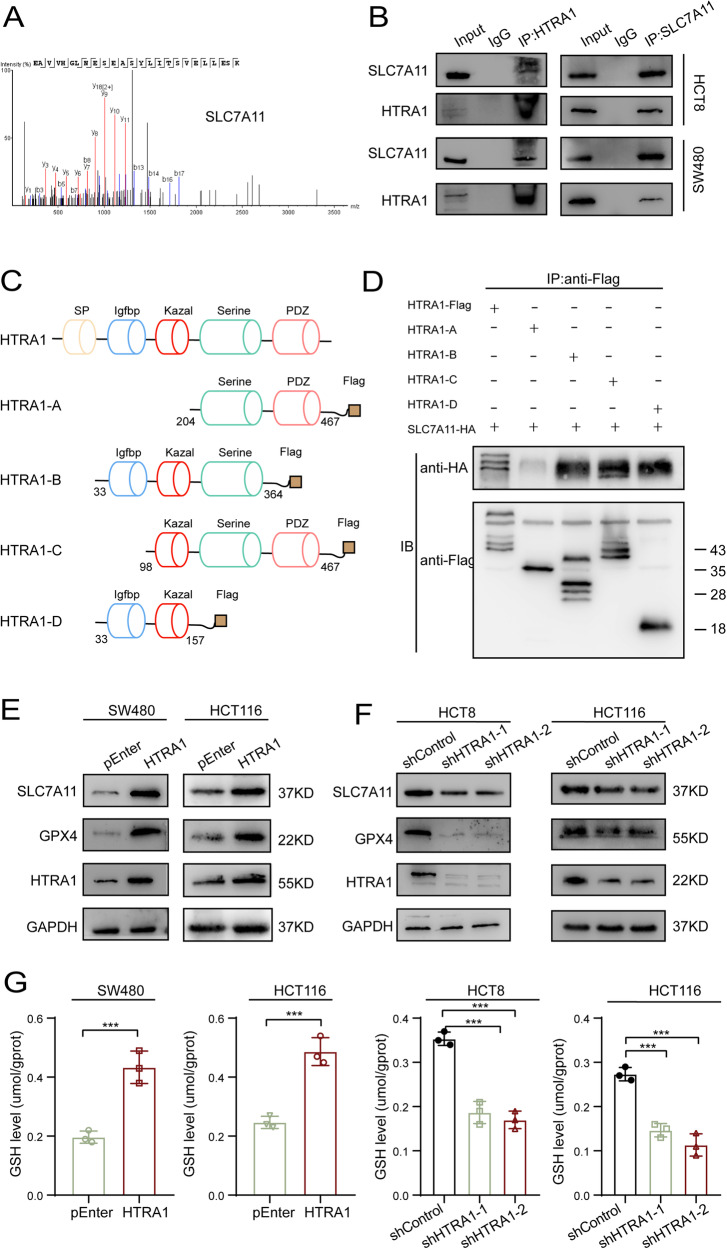
HTRA1 interacts with SLC7A11 and promotes its expression. **A** Mass spectropraphy analysis indicates that SLC7A11 is a HTRA1-interacting protein. **B** Co-IP verified the interaction between HTRA1 and SLC7A11. **C** The schematic structure of HTRA1 and truncated HTRA1s. **D** Western blotting assays demonstrate the binding domains of HTRA1 with SLC7A11 in the 293T cells. **E**, **F** Western blotting assays show the regulation of HTRA1 on SLC7A11. **G** The effects of HTRA1 on the GSH levels in HCT8 and HCT116 cells. All the data represent the mean ± SD at least three independent experiments. **P < 0.01 and ***P < 0.001. Differences were tested using an unpaired two tailed Student’s *t* test (**G**).

To explore the molecular mechanism underlying HTRA1-mediated ferroptosis, immunoprecipitation was performed using HTRA1 antibody, and the HTRA1-interacting proteins were confirmed by mass spectrometry. SLC7A11, a key protein related to polyunsaturated fatty acid synthesis, was screened out as a candidate involving in HTRA1-mediated ferroptosis (Fig. 6A). Further co-immunoprecipitation experiments confirmed a intracellular interaction between HTRA1 and SLC7A11 (Fig. 6B). Several truncated HTRA1s with Flag-tag were constructed, and the specific interaction site between HTRA1 and SLC7A11 was elucidated (Fig. 6C). To further clarify the exact structural domain of HTRA1 that is essential for binding with SLC7A11, we transfected 293T cells with the truncated HTRA1s together with SLC7A11. SLC7A11 was undetectable in Co-IP assay only when the Kazal structural domain of HTRA1 was absent, suggesting that HTRA1 may bind to SLC7A11 through the Kazal structural domain (Fig. 6D). Interestingly, the upregulated expression of SLC7A11 and GPX4 could be detected by western blot (Fig. 6E). Consistently, knockdown of HTRA1 significantly suppressed the expression SLC7A11 and GPX4 at translational level (Fig. 6F). Since the cystine/glutamate antitransporter SLC7A11 is essential for GSH synthesis as a major co-factor in the endogenous antioxidant system, we investigated the effects of HTRA1 on intracellular GSH and GSSH levels. The results showed that overexpression of HTRA1 could increase the levels of GSH and GSSH in CRC cells, while knockdown of HTRA1 could significantly decrease the levels of GSH and GSSH in CRC cells (Fig. 6G and Supplementary Fig. S6A).

### SLC7A11 mediates the HTRA1-inhibited ferroptosis in colorectal cancer cells

**Fig. 7:**
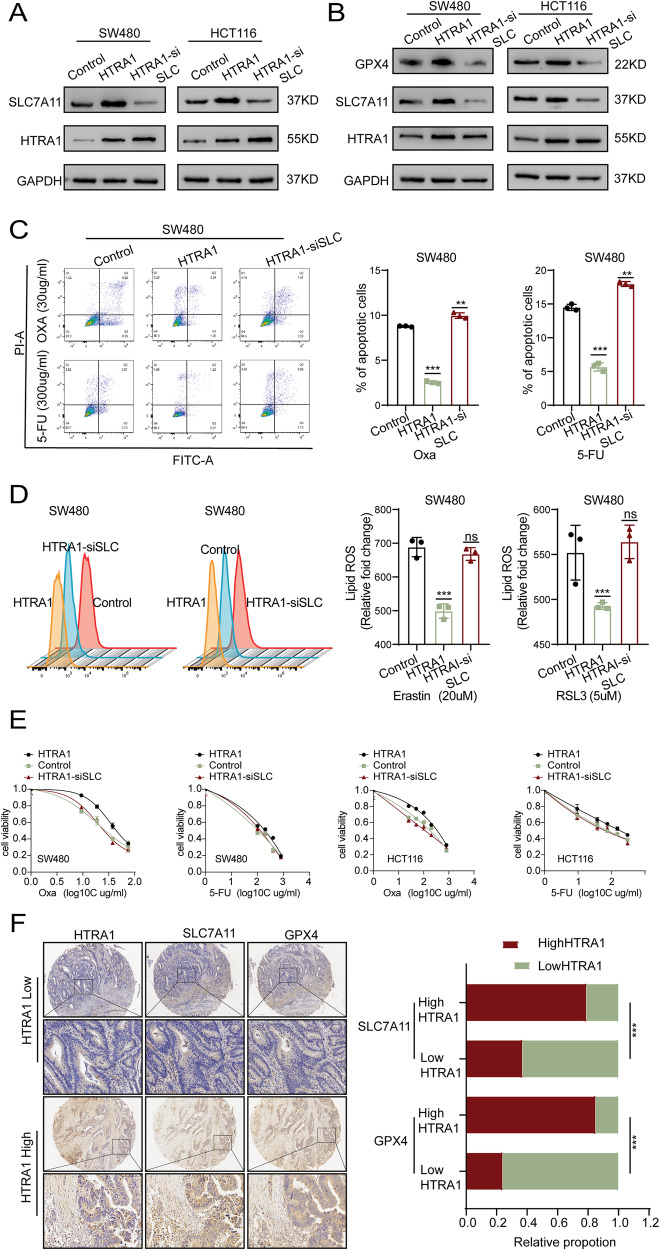
The mediatory role of SLC7A11 in HTRA1-regulated ferroptosis. **A** Western blotting assays show the effect of siSLC7A11 on the expression of HTRA1. **B** Western blotting assays demonstrate the effect of siSLC7A11 on the expression of GPX4 in HTRA1 overexpression CRC cells. **C** Flow cytometry assays demonstrate the effect of siSLC7A11 on the apoptosis of HTRA1 overexpression SW480 cells treated with 5-FU and oXA. **D** Flow cytometry assays show the regulation of siSLC7A11 on the lipid ROS levels in HTRA1 overexpression cells. **E** The effects of siSLC7A11 on the cell viability of HTRA1 overexpression cells treated with Erastin and RSL3. **F** The left panel shows the immunohistochemical analyses of HTRA1, SLC7A11 and GPX4. The right panel shows the correlation of HTRA1 with SLC7A11 and GPX4. All the data represent the mean ± SD at least three independent experiments. **P < 0.01 and ***P < 0.001. Differences were tested using an unpaired twotailed Student’s *t* test (**C**, **D**) and Chi-square test was performed to determine the correlation (**F**).

To verify whether SLC7A11 mediated the HTRA1-inhibited ferroptosis in CRC cells, we transfected HTRA1 overexpression CRC cells with si-SLC7A11 plasmid in SW480 and HCT116 cells (Fig. 7A). Western blot assays indicated that si-SLC7A11 could suppress the expression of GPX4 in HTRA1 overexpression CRC cells (Fig. 7B). Flow cytometry assays showed that si-SLC7A11 reversed the effect of HTRA1 on apoptosis (Fig. 7C and Supplementary Fig. S7A). Moreover, flow ytometry analysis showed that the suppressed ROS levels in CRC cells caused by HTRA1 were also restored detected (Fig. 7D and Supplementary Fig. S7B). Drug sensitivity assays also confirmed the mediatory role of SLC7A11 in HTRA1-enhanced resistance to L-OHP and 5-FU in SW480 and HCT116 cells (Fig. 7E).

Finally, we examined the correlation between HTRA1, SLC7A11, and GPX4 using tissue microarray (TAM) of clinical CRC tissues. The results showed that high expression of HTRA1 was correlated with the high expression of SLC7A11 and GPX4 (Fig. 7F).

## Discussion

Chemoresistance is a complex process involving multiple genes and signaling pathways. Several vital events including decreased drug uptake, increased drug efflux, enhanced DNA repair, and inhibition of apoptosis are associated with chemoresistance. In addition, increasing evidence suggests that the intracellular ROS imbalance-mediated oxidative stress defense is also closely related to chemoresistance [4]. Chemotherapeutic drugs can induce the production and accumulate of ROS in tumor cells to cause oxidative stress, which leads to DNA damage, lipid oxidation, and a series of alterations in the biological functions of proteins, thus, ultimately leading to tumor cell damage and death. However, tumor cells will develop chemoresistance by altering their metabolic microenvironment to inhibit ROS formation, or enhancing oxidative stress defense and tolerance [5]. Ferroptosis is a new form of cell death distinguished from apoptosis, necrosis, and autophagy, and it is caused by iron-dependent lipid ROS accumulation [6]. With the hlep of divalent iron, ester oxygenase can catalyze the peroxidation of polyunsaturated fatty acid phospholipids (PL-PUFA) in cell membrane, thus elevating lipid ROS levels to induce cell death. Meanwhile, a reduced expression of GPX4 and GSH can also be detected in CRC cells [7, 8]. In recent years, lipid ROS imbalance-mediated cellular ferroptosis is thought to be closely related to tumor drug resistance, but the specific mechanism by which tumor cells inhibit ferroptosis and induce chemoresistance is not clear.

HTRA1 has been screened out successfully as a progression-related gene for CRC. As a member of HTRA serine protease family, HTRA1 also obtains heat shock protein properties. It is abnormally expressed in a variety of tumors, and its bi-directional regulation has been detected in different cancer types [14–17]. However, studies on HTRA1 were mainly focused on its effect on tumor progression, the functional mechanism of of HTRA1 in colorectal cancer cells has not been clarified. In consistent with the result of bioinformatic analyses, the IHC staining of TMA has also demonstrated the overexpression of HTRA1 in CRC tissues, and it is mainly localized in the cytoplasm of CRC cells. HTRA1 could stimulate the growth of CRC cells both in vitro and in vivo. Since HTRA1 was positively correlated with the poor prognosis of CRC patients with chemotherapy, we further conducted the drug sensitivity experiments, and confirmed the inhibition of HTRA1 on chemosenitivity. To reveal the mechanism underlying the HTRA1 mediated chemoresistance, HTRA1-interacting proteins were predicted using the STRING database, and the following enrichment analyses indicated the involvement of oxidative stress-related pathways. Hence, we proposed that ferroptosis might contribute to the HTRA1-mediated chemoresistance. The apoptosis inhibitor Z-VAD-FMK, and ferroptosis inhibitor Ferrostatin-1 were used to exclude other types of cell death. Moreover, compared to HTRA1 overexpression CRC cells, the control cells have a wrinkled volume, increased mitochondrial membrane density, loss of mitochondrial cristae, and rupture of the outer mitochondrial membrane. The results demonstrated a vital role of ferroptosis in HTRA1-stimulated chemoresistance.

To further explore molecular mechanisms underlying the HTRA1-stimulated chemoresistace, the immunoprecipitation was conducted and SLC7A11 was considered as a HTRA1-interacting protein. SLC7A11 is a key protein related to polyunsaturated fatty acid synthesis, and co-immunoprecipitation experiments confirmed a direct intracellular interaction between HTRA1 and SLC7A11. The further co-immunoprecipitation assay conducted by truncated HTRA1s and SLC7A11 suggested that HTRA1 might bind to SLC7A11 directly through the Kazal structural domain. Interestingly, we also find a stimulatory effect of HTRA1 on the expression of SLC7A11 and GPX4 by western blot assay. However, we haven’t revealed the detailed mechanism underlying the regulatory role of HTRA1 on SLC7A11 and GPX4. It is probable that HTRA1 could increase the stability of the two proteins as a heatshock protein. SLC7A11 is a cystine/glutamate antiporter, and we found that overexpression of HTRA1 could increase the GSH levels in CRC cells to regulate ferroptosis. In addition, there is a 22 amino acids secretory peptide sequence in the N-terminal of HTRA1, which means HTRA1 might also function as a secretory protein to influence CRC chemoresistance. But we mainly focus on the functional role of HTRA1 in CRC cells in this manuscript.

It has been found that interference of different pathways regulating ferroptosis and modulating the tolerance of tumor cells to oxidative stress can affect chemoresistance. Clinical application of the iron chelator deferoxamine (DFO) on cervical cancer cells could enhance their sensitivity to oxaliplatin [18]. Activation of NRF2, a nuclear transcription factor-associated with oxidative stress, up-regulated the expression of MT-1G and promoted sorafenib resistance through inhibiting ferroptosis in hepatocellular carcinoma cells [19]. Inhibition of the KEAP1-NRF2 signaling pathway stimulated the occurence of ferroptosis and reverse the resistance of cisplatin [20]. In addition, the combination of ferroptosis inducers with chemotherapeutic agents has significant synergistic effects against tumors and might be a potential therapeutic strategy in various of tumors. For example, the ferroptosis inducer Erastin could significantly enhance the therapeutic effect of cisplatin against ovarian cancer [21]. Erastin could also significantly enhance the anticancer activity of cytarabine and doxorubicin in HL60 cells, and the ferroptosis it induced could reverse the chemoresistace in acute myeloid leukemia cells [22, 23]. In addition, some clinical drugs can induce ferroptosis, such as sorafenib, salazosulfapyridine and artesunate, has been used as preclinical and clinical adjuvant chemotherapeutic agents for tumor treatment [24]. Therefore, further elucidation of ferroptosis and its mechanism in chemotherapy tolerance may create new opportunities for reversing chemoresistacne, and targeting key genes that regulate ferroptosis is likely to be a new means of improving chemotherapy resistance in tumor therapy. In this study, we did not test the effects of combination therapy of HTRA1 and ferroptosis inhibitors on CRC in vivo. As there are peptides and small molecules inhibitors for HTRA1, it is a potential therapeutic traget for CRC treatment, especially chemoresistace.

In a summary, we screened HTRA1 out as a CRC progression-related gene and studied its role on the progression of CRC cells. We also revealed a stimulatory role of HTRA1 in chemoresistance in a ferroptosis-dependent manner. We suggested that HTRA1 might be a therapuetic target in CRC treatment.

## Materials and methods

### Cell culture and treatment

Human embryonic kidney (HEK) 293T cells, normal human colon epithelial NCM460 cells and CRC cells including HCT8, RKO, CACO2, SW620, SW480, HCT116 and DLD1 were purchased from the Cell Bank of the Chinese Academy of Sciences. These cells were cultured in RPMI 1640 (Hyclone, China) supplemented with 10% Fetal Bovine Serum (FBS, Gibco-BRL, Invitrogen). They were cultured in a humidified 5% CO_2_ incubator at 37 °C, and all the experiments were carried out during their logarithmic growth phase. Chemotherapeutic agents such as 5-fluorouracil (5-FU, Selleck, China) and oxaliplatin (L-OHP, MCE, China) was administrated to CRC cells at a final concentration of 800 μg/ml and 6 mg/ml, respectively, to investigate the chemosensitivity of CRC cells.

The shRNA and siRNA targeting HTRA1 and SLC7A11 were synthesized by Tsingke Biotech (Beijing, China). The wild-type HTRA1 overexpression vector with FLAG tag (pEnter-HTRA1) was purchased from Generay Biotech (Shanghai, China). All plasmids, siRNAs and shRNAs were transfected into CRC cells using Lipofectamine 3000 (Invitrogen, USA). HTRA1 stable overexpressing CRC cells were obtained by antibiotic screening after transfection. All siRNA and shRNA sequences are listed in Supplementary Table S1.

### Clinical human CRC samples

Fresh clinical human CRC tissues and tumor specimens for tissue microarray (TMA) were collected from affiliated Hospitals of Southern Medical University. A diagnosis of primary CRC had been made before carrying out surgery between 2017 and 2022, and none of the patients had received neoadjuvant therapy. The study was approved by the Biomedical Research Ethics Committee of Shunde hospital Southern Medical University (No. KYLS20231108), and the informed consent was obtained from all the patients. In all aspects, the study is complied with the Declaration of Helsinki.

### Real-time quantitative PCR

Total cellular RNAs was extracted using TRIzol reagent (Takara,China), and 1 μg of which was used to perform cDNA synthesis. The real-time qPCR reaction was conducted by 7500 Fast Real-Time PCR System (Roche, China) using SYBR Green PCR Master Mix (Takara, China). The PCR cycling proflies was 95 °C for 30 s, followed by 40 cycles of denaturation at 95 °C for 5 s, and annealing at 60 °C for 30 s. A post-amplification melting curve was performed by heating the PCR products to 95 °C. The mRNA levels of target genes were normalized to that of the housekeeping gene GAPDH and calculated by the 2^−ΔΔCT^ method. Primers used in this study are listed in Supplementary Table S1.

### Immunohistochemistry

Paraffin-embedded tissue sections were incubated in a 60 °C oven for 2 h. Then, they were deparaffinized with xylene and rehydrated with alcohol. Endogenous peroxidase was blocked with 3% hydrogen peroxide for 10 min. Antigen repair was performed in 10 mM citrate buffer (pH 6.0) in an autoclave with high pressure for 5 min. The tissue sections were then blocked with 5% BSA for 1 h and incubated overnight at 4 °C with primary antibodies. The next day, tissue sections were incubated with HRP-labeled secondary antibody for 1 h and stained with DAB substrate for 5 min, which was followed by hematoxylin staining.

### Western blot analysis

CRC cells were harvested and washed with pre-cooled PBS, before they were lysed in RIPA lysis buffer supplemented with protease inhibitor mixture and PMSF. The lysates were centrifuged at 12,000 × *g* for 30 min at 4 °C. Protein concentration was measured by BCA assay (Invitrogen, China), before they were separated by SDS-PAGE and transferred to a PVDF membrane. After blocking in TBST buffer with 5% skimmed milk, the membranes were incubated with primary antibodies such as HTRA1 (PA5-11412), GPX4 (67763-1-lg) and SLC7A11 (26864-1-AP), at 4 °C overnight. The next day, the membranes were washed and incubated with HRP-labeled secondary antibodies for 1 h at room temperature. The visualization was performed by 4200SF detector (Tanon, China) after staining by ECL substrates, according to the manufacturer’s instructions.

### Cell viability assay and transwell migration assay

CRC cells were inoculated into a 96-well plate and growth to the logarithmic phase. The CCK-8 solution (Dojindo,China) was added to each well and incubated at 37 °C for 2 h. Absorbance at 450 nm were measured on a spectrophotometer, and cell viability was quantified.

Cell migration was examined by the transwell assay. The lower transwell chamber was filled with 750 µl of RPMI-1640 containing 10% FBS, while the upper one was filled with 200 µl serum-free RPMI-1640 inoculated with 2 × 10^4^ CRC cells. The transwell chambers were incubated at 37 °C for 24 h before the migrated cells were fixed with 4% paraformaldehyde and stained with 1% crystal violet. Images were captured using a microscope and the number of migrated cells were counted.

### Immunoprecipitation

CRC cells were washed with ice-cold PBS and lysed in immunoprecipitation buffer containing 20 mM Tris-HCl (pH 7.4), 150 mM NaCl, 1 mM EDTA, 1% Triton X-100, a protease inhibitor mixture and PMSF. Cell lysates were centrifuged at 12,000 × *g* for 30 min at 4 °C, and then incubated with Protein A/G beads at 4 °C overnight. Immunoprecipitates were washed thoroughly with IP wash buffer (10 mM Tris, pH 7.5, 150 mM NaCl, 1 mM EDTA, and 0.2% Triton X-100) and immunoblotted with the antibodies.

### Apoptosis analysis

Apoptosis was detected using Annexin V-FITC/Annexin V-APC and propidium iodide (PI) double staining (KEYGEN-KGA107, KEYGEN-KGA1030, China). Cells were inoculated to a six-well plate at a concentration of 1.2 × 10^5^/well and incubated overnight to allow them adhering to the plates. Then the culture medium was removed and changed to fresh medium with drugs and incubated for another 24 h. The cells were then digested with EDTA-free trypsin and washed twice with pre-cooled PBS. Resuspended the cells in 100 µl binding buffer and stained with 1 µl V-FITC/Annexin V-APC and 1 µl of PI working solutions for 15 min at room temperature in dark places. All flow cytometric analyses were performed using a FACSCalibur flow cytometer.

### Lipid Reactive Oxygen Species (ROS) detection

Lipid ROS levels were estimated by the DCFH-DA fluorescent dye. The cells was incubated with DCFH-DA fluorescent dye in the medium at 37 °C for 30 min. The results were analyzed using FlowJo V10 software after Flow cytometry analysis.

### Measurement of Malondialdehyde (MDA) and Glutathione (GSH)

CRC cells were inoculated in 6-well cell culture plates (Corning, China). After the cells were harvested, protein concentration was measured using a NanoDrop 2000 spectrophotometer (Thermo, Waltham, MA, USA), and then the MDA level in CRC cells were measured using a lipid peroxidation MDA assay kit (Abcam, UK; ab118970) according to the instructions. MDA levels were determined as the ratio of MDA levels to protein concentration.

Relative GSH level were calculated in all groups using the reduced Glutathione (GSH) content assay kit (BC1170-50T/48S, Solarbio) according to the manufacturer’s instructions.

### Subcutaneous xenograft mouse model

The male BALB/c-nu/nu mice at 4 weeks old weighing 18–22 g were purchased from Guangdong Medical Laboratory Animal Center. These mice were maintained in a specific pathogen-free environment. All animal experiments were conducted in accordance with the guidelines of the Institutional Animal Ethics Committee of the Southern Medical University Laboratory Animal Center (No. SMUL202310035). About 5 × 10^6^ CRC cells were dissolved in 100 μl of PBS, and injected subcutaneously into nude mice. At the end of the third week, mice were executed and solid tumors were dissected.

### Statistical analysis

All experiments, except for those involving mice, were performed in at least three independent biological replicates, with technical replicates for each experiment. Data are expressed as the mean ± SD. For data with a normal distribution, unpaired or paired two-tailed Student’s *t*-tests were used to compare the significance of differences between two groups of independent samples. For the in vivo experiments related to animals, the number of biological replicates is 4 or 5. Animals were allocated to control experimental groups using a blinding and randomization method. The correlation between HTRA1 expression and clinicopathological factors in CRC patients was assessed using chi-square test. Statistics were performed by GraphPad Prism 8.0. A *P*-value less than 0.05 was considered statistically significant. All *P*-values are indicated in the figures (#*P* > 0.05; *P < 0.05; **P < 0.01;***P < 0.001).

### Supplementary information

Supplementary material

WB raw data

## Data Availability

The datasets generated and/or analyzed during the current study are not publicly available but are available from the corresponding author upon reasonable request.
