# Supplementary material for: HTRA1 interacts with SLC7A11 to modulate colorectal cancer chemosensitivity by inhibiting ferroptosis
Source: Cell Death Discov. 2024 May 13;10:228. doi: 10.1038/s41420-024-01993-6 (PMC11091184; doi:10.1038/s41420-024-01993-6)
Supplement: Supplementary file 1 — Supplementary material [file 41420_2024_1993_MOESM1_ESM.pdf]

Supplementary Figures and Table

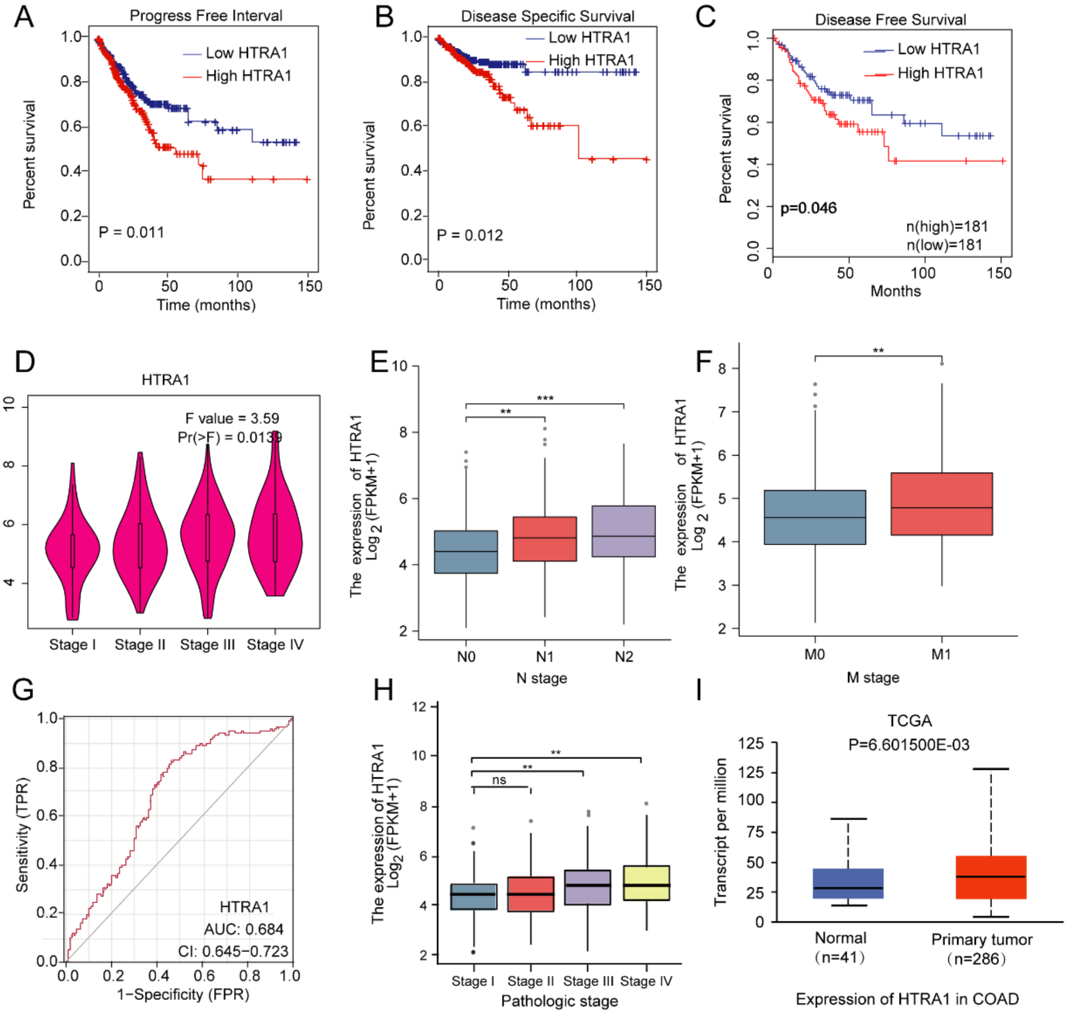

Figure S1. The correlation between the expression of HTRA1 and the prognosis of CRC patients.

A-C. The correlation between the expression of HTRA1 and the Progress Free Interval, Disease Specific Survival and Disease Free Survival of CRC patients. D-F. The correlation between the expression of HTRA1 and the TNM stages of CRC. G. The ROC analysis shows the predictive potential of HTRA1 in evaluating the prognosis of CRC patients. H. The correlation between the expression of HTRA1 and the pathologic stage of CRC using the TCGA database. * p < 0.05,** p < 0.01, ** p < 0.001.

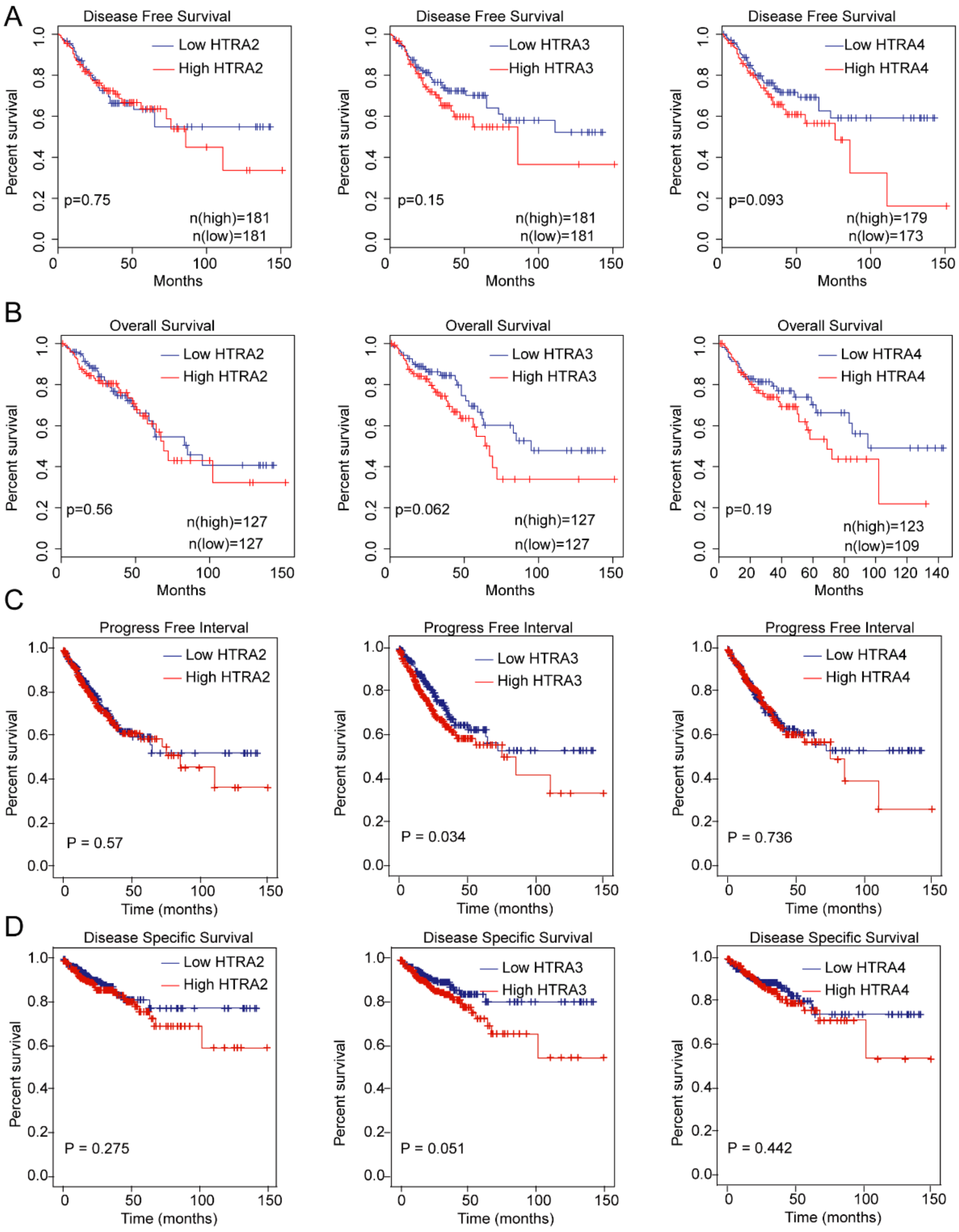

Figure S2. The relationship between the HTRA family members and the prognosis of CRC patients.

A-D. The Relationship between the expression of HTRA family members including HTRA2, HTRA3 and HTRA4, and the prognosis of CRC patients including Disease Free Survival, Overall Survival, Progress Free Interval and Disease Specific Survival in CRC. * $p < 0.05$,** $p < 0.01$, ** $p < 0.001$.

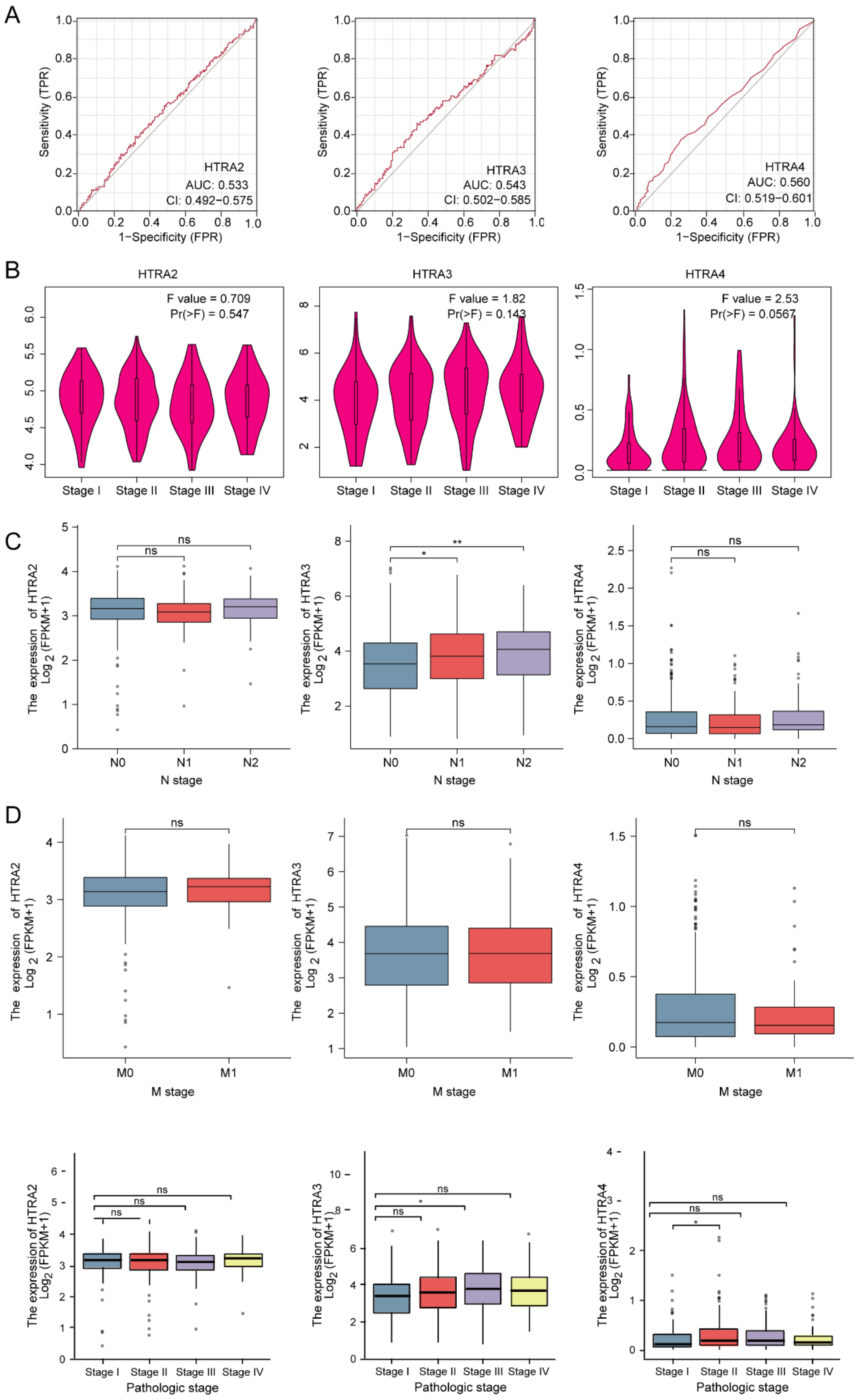
A
Sensitivity (TPR)
1−Specificity (FPR)
HTRA2
AUC: 0.533
CI: 0.492−0.575
HTRA3
AUC: 0.543
CI: 0.502−0.585
HTRA4
AUC: 0.560
CI: 0.519−0.601
B
HTRA2
F value = 0.709
Pr(>F) = 0.547
HTRA3
F value = 1.82
Pr(>F) = 0.143
HTRA4
F value = 2.53
Pr(>F) = 0.0567
Stage I
Stage II
Stage III
Stage IV
C
The expression of HTRA2 Log 2 (FPKM+1)
The expression of HTRA3 Log 2 (FPKM+1)
The expression of HTRA4 Log 2 (FPKM+1)
N0
N1
N2
N stage
ns
*
**
D
M0
M1
M stage
Pathologic stage

Figure S3. The expression and predictive potential of HTRA family members on the prognosis of CRC patients.

A. The ROC analysis shows the predictive potential of HTRA2, HTRA3 and HTRA4 on the prognosis of CRC patients, B-D. The association between the expression of HTRA family members and TNM stages in CRC. E. The association between the expression of HTRA family members and the pathologic stages of CRC. * $p < 0.05$,** $p < 0.01$, ** $p < 0.001$.

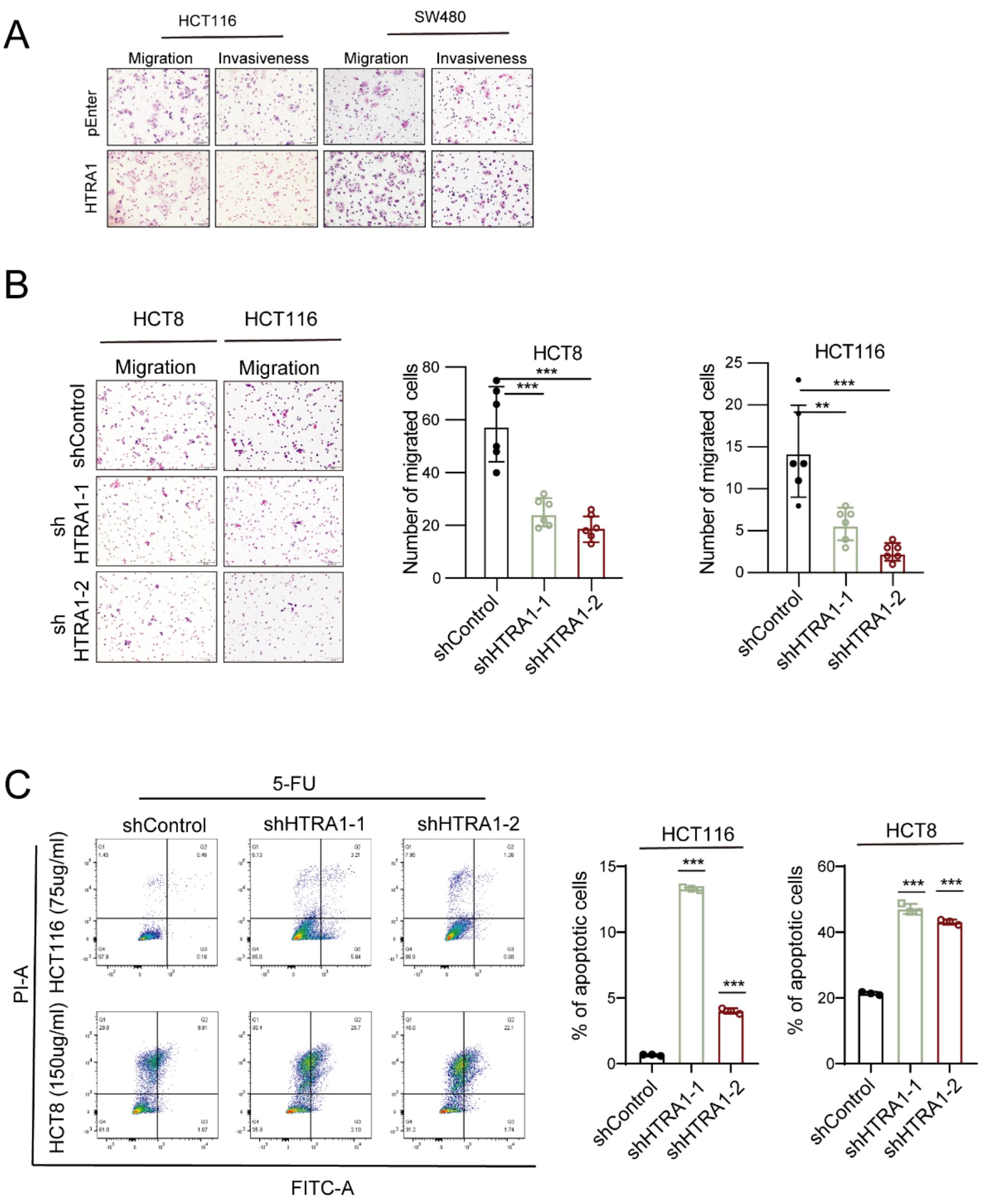

Figure S4. The effects of HTRA1 on the proliferation and chemoresistance of CRC cells.

A. Transwell assays demonstrate the effect of HTRA1 on the invasive and migratory ability of SW480 and HCT116 cells. B. Transwell assay shows the effects of HTRA1 on the migratory ability of HCT8 and HCT116 cells. C. Flow cytometry analysis shows the effect of HTRA1 on the apoptosis of CRC cells after 5-FU and oXA treatment. All the Data represent the mean ± SD at least three independent

experiments. **$P < 0.01$ and ***$P < 0.001$. Differences were tested using an unpaired twotailed Student's t-test (B-C).

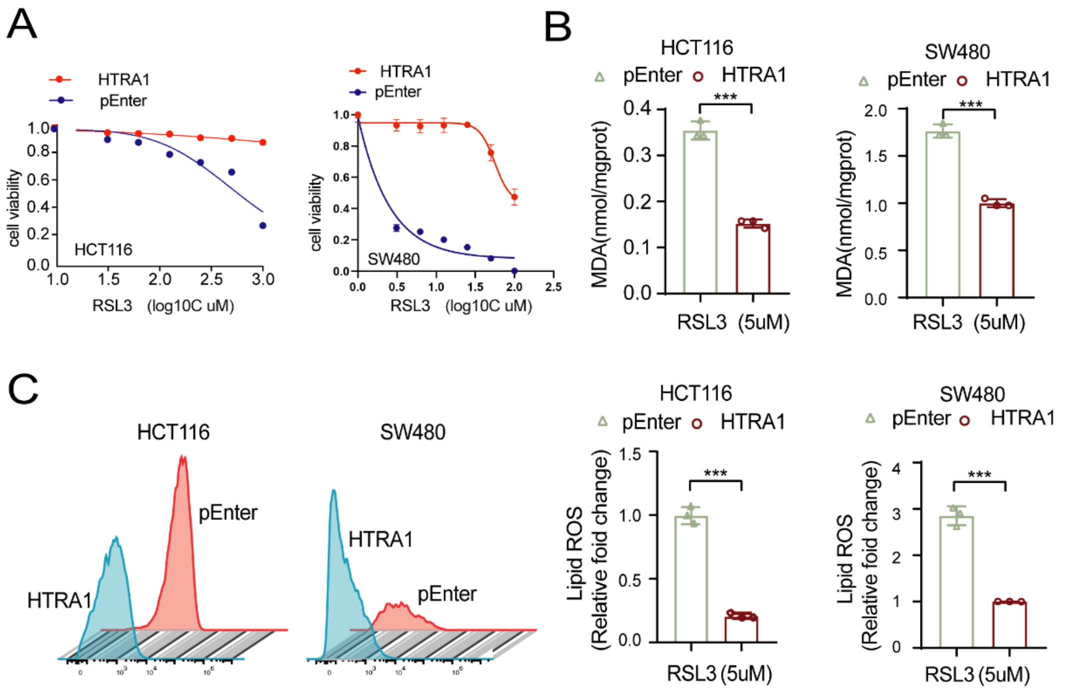

Figure S5. HTRA1 influences the ferroptosis of CRC cells.

A. The effect of RSL3 on the cell viability of control and HTRA1 overexpression CRC cells. B. The MDA levels were detected in control and HTRA1 overexpression CRC cells treated with RSL3 for 24 h. C, Flow assay shows the regulation of HTRA1 on the lipid ROS in CRC cells. All the Data represent the mean ± SD at least three independent experiments. $^{**}P < 0.01$ and $^{***}P < 0.001$. Differences were tested using an unpaired twotailed Student's t-test (B-C).

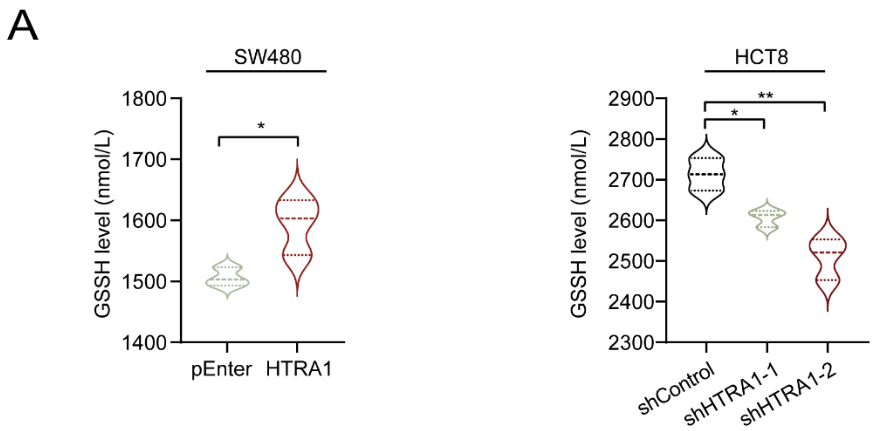

Figure S6. ELISA detects the effects of HTRA1 on GSSH uptake in CRC cells.

All the data represent the mean ± SD at least three independent experiments. **P < 0.01 and ***P < 0.001. Differences were tested using an unpaired twotailed Student's t-test (A-B).

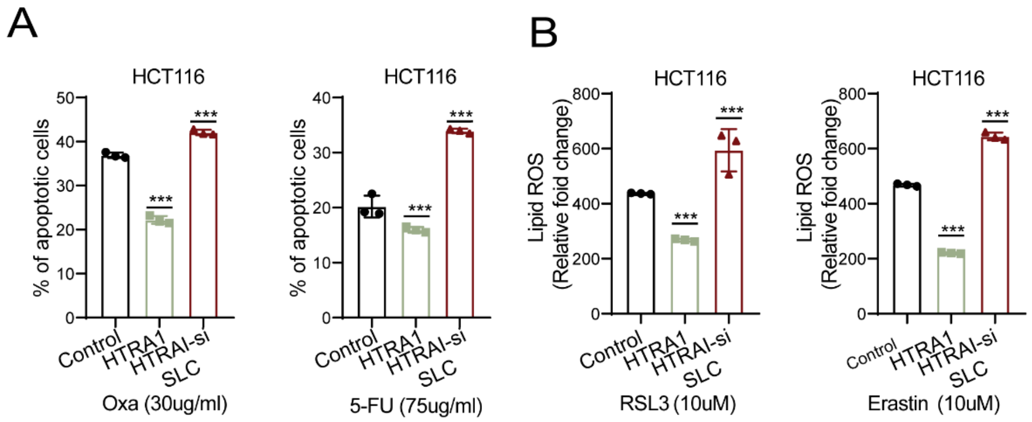

Figure S7. HTRA1 inhibits the ferroptosis in CRC cells mainly by upregulating the expression of SLC7A11 levels.

A Flow cytometry assays demonstrate the effect of siSLC7A11 on the apoptosis of HTRA1 overexpression HCT116 cells treated with 5-FU and oXA. B. Flow cytometry assays demonstrate the effect of siSLC7A11 on the HTRA1-regulated lipid ROS levels in CRC cells. All the data represent the mean ± SD at least three independent experiments. $^{**}P < 0.01$ and $^{***}P < 0.001$. Differences were tested using an unpaired twotailed Student's t-test (A-B).

Supplementary Table S1. RT-qPCR primer and shRNA sequences for human genes

	Gene	Sequence
**RT-qPCR**	**HTRA1**	**Forward:** 5'-CAGTCACCACTGGGATCGTC-3'<br>**Reverse:** 5'-AATTGATGATAGCGTCTGTCTGAA-3'
	**GAPDH**	**Forward:** 5'-GGAGCGAGATCCCTCCAAAAT-3'<br>**Reverse:** 5'-GGCTGTTGTCATACTTCTCATGG-3'
**shRNA**	**HTRA1**	**shRNA1:** 5’-CGGTGAAGTGATTGGAATTAA-3’
**shRNA**	**HTRA1**	**shRNA2:** 5’-GAAGTATATTGGTATCCGAAT-3’
