# Supplementary material for: HTRA1 interacts with SLC7A11 to modulate colorectal cancer chemosensitivity by inhibiting ferroptosis
Source: Cell Death Discov. 2024 May 13;10:228. doi: 10.1038/s41420-024-01993-6 (PMC11091184; doi:10.1038/s41420-024-01993-6)

Figure1

1E

HTRA1

GAPDH

HTRA1

GAPDH

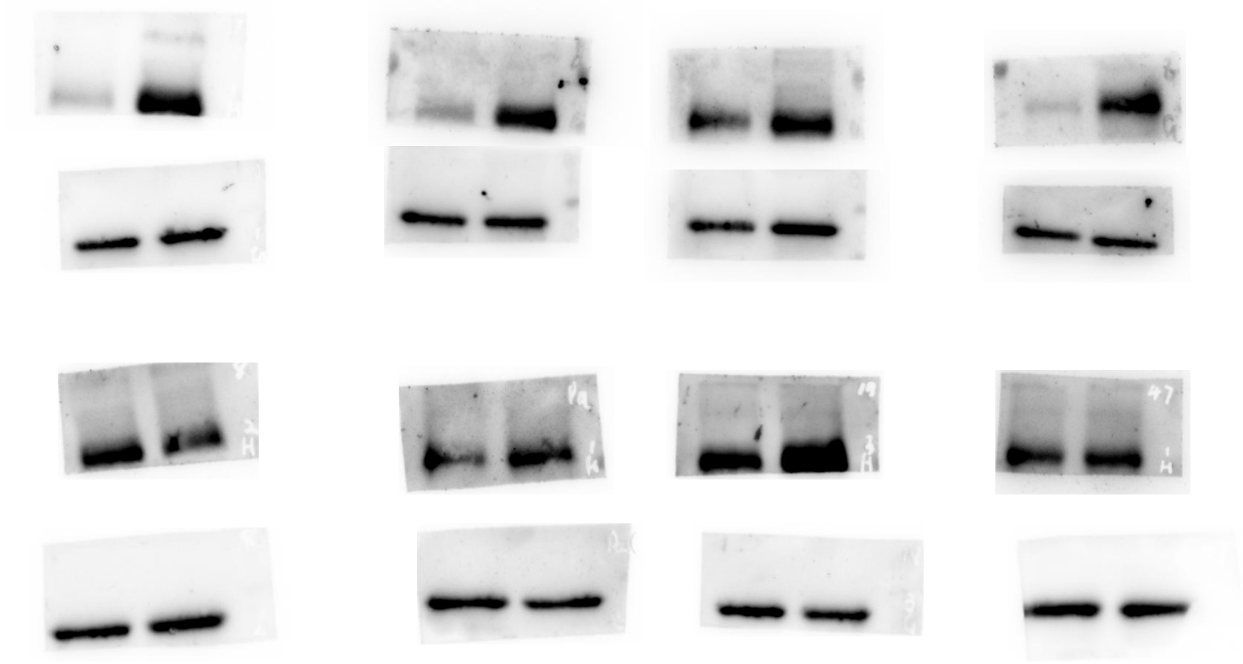

1F

HTRA1

GAPDH

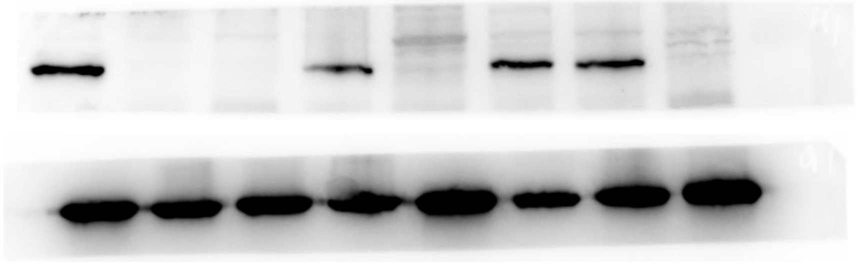

Figure2

1A

HTRA1

GAPDH

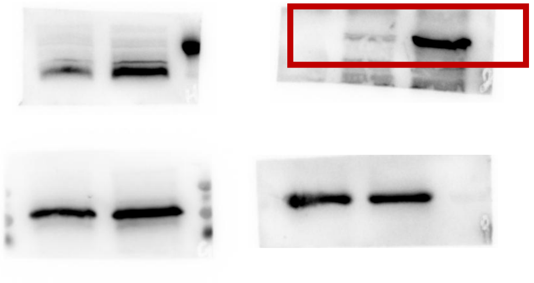

Figure3

1A

HTRA1

GAPDH

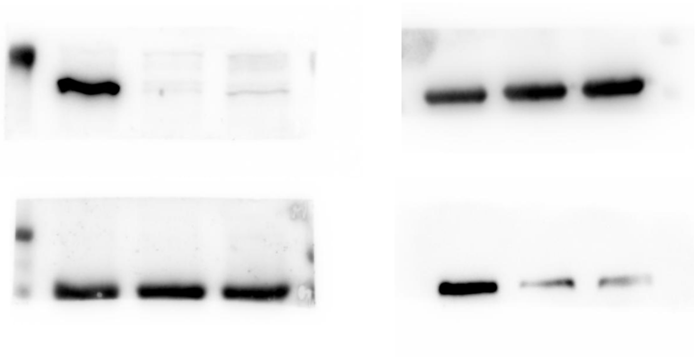

Figure6

6B

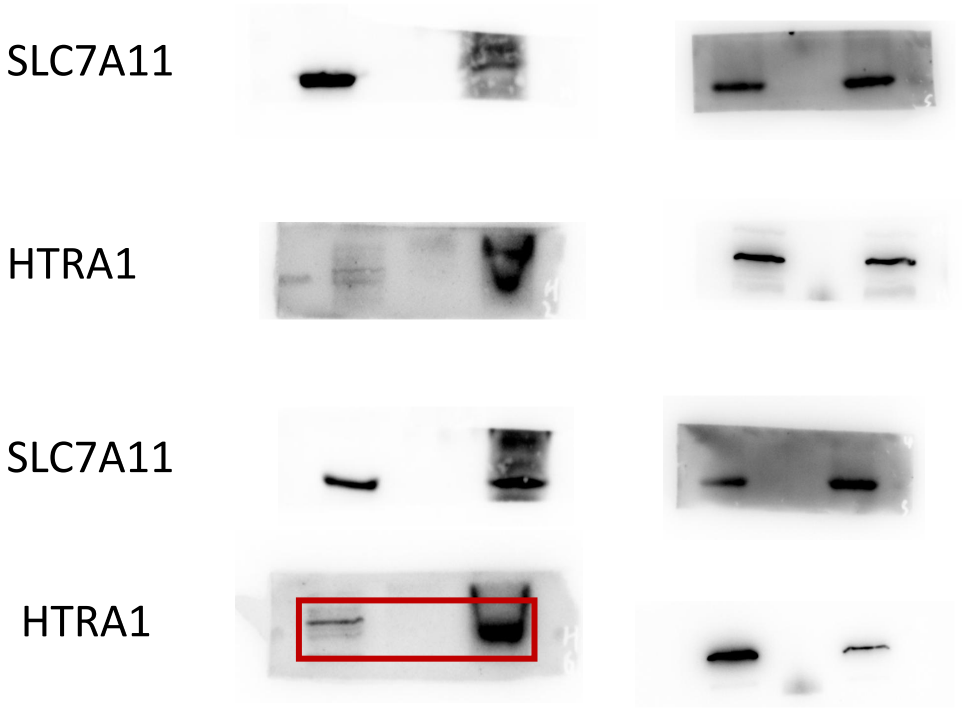

6D

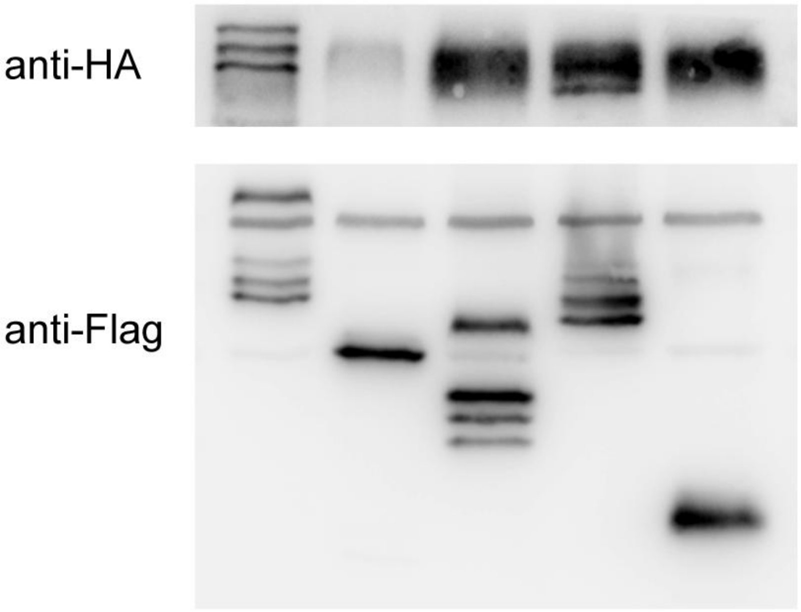

Figure6

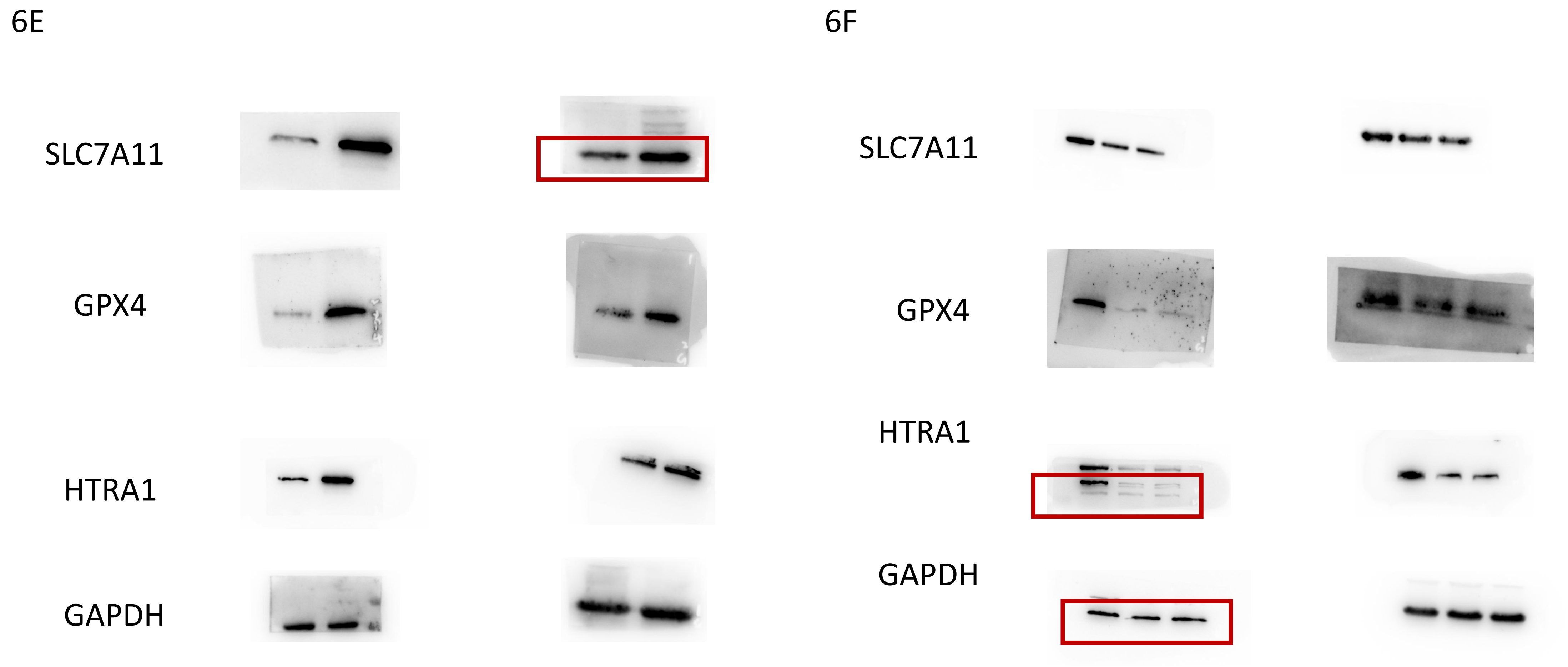

Figure7

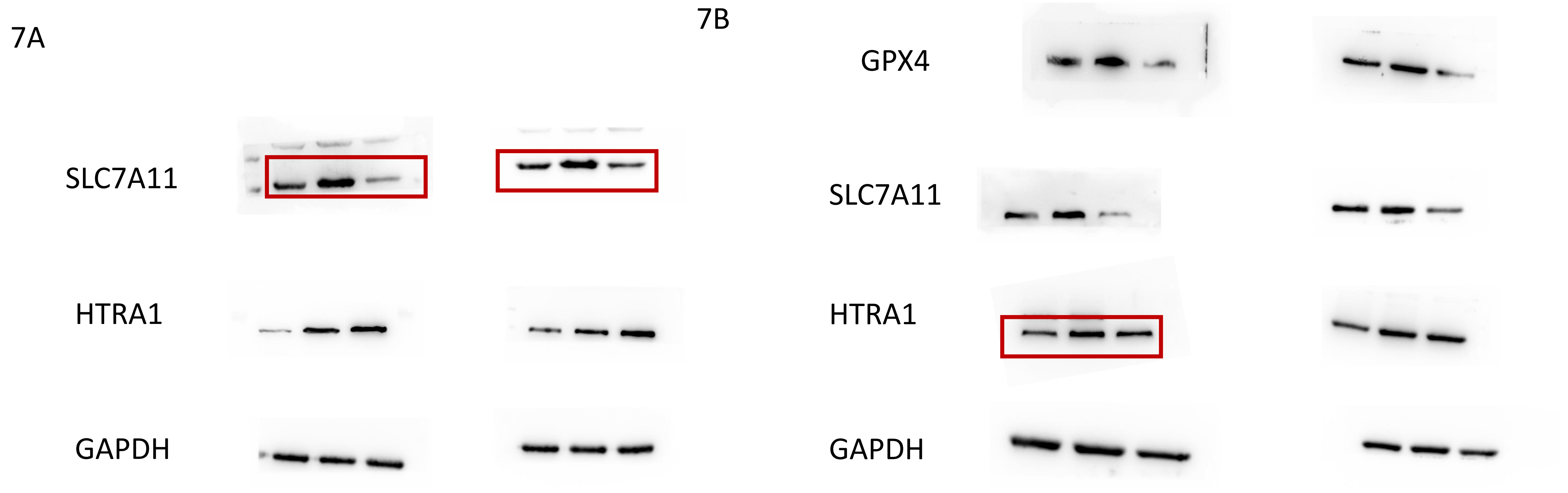

Supplement: Supplementary file 2 — WB raw data [file 41420_2024_1993_MOESM2_ESM.pdf]
